# Serum microRNA signatures and metabolomics have high diagnostic value in hepatocellular carcinoma

**DOI:** 10.18632/oncotarget.22224

**Published:** 2017-11-01

**Authors:** Hai-Ning Liu, Hao Wu, Yan-Jie Chen, Yu-Jen Tseng, Enkhnaran Bilegsaikhan, Ling Dong, Xi-Zhong Shen, Tao-Tao Liu

**Affiliations:** ^1^ Department of Gastroenterology, Zhongshan Hospital of Fudan University, Shanghai 200032, China; ^2^ Shanghai Institute of Liver Diseases, Zhongshan Hospital of Fudan University, Shanghai 200032, China

**Keywords:** liver neoplasms, diagnostic test, meta-analysis, microRNA, metabolomics

## Abstract

**Background:**

Many new diagnostic biomarkers have been developed for hepatocellular carcinoma (HCC). We selected two methods with high diagnostic value, the detection of serum microRNAs and metabolomics based on gas chromatography/mass spectrometry (GC/MS), and attempted to establish appropriate models.

**Methods:**

We reviewed the diagnostic efficiencies of all microRNAs identified by previous diagnostic tests. Then we chose appropriate microRNAs to validate the diagnostic efficiencies, and determined the optimal combination. We included 66 patients with HCC and 82 healthy controls (HCs) and detected the expression of the microRNAs. GC/MS analysis was performed, and we used three multivariate statistical methods to establish diagnostic models. The concentration of alpha feto-protein (AFP) was determined for comparison with the novel models.

**Results:**

82 published studies and 92 microRNAs were ultimately included in this systematic review. Seven microRNAs were selected for further validation of their diagnostic efficiencies. Among which, miR-21, miR-106b, miR-125b, miR-182 and miR-224 had a significantly different expression in HCC patients. The combination of miR-21, miR-106b and miR-224 had the highest area under the curve (AUC) at 0.950 with a sensitivity of 80.3% and a specificity of 92.7%. The GC/MS analysis exhibited an excellent diagnostic value and the AUC reached 1.0. In comparison, the AUC of the traditional biomarker, AFP, was 0.755.

**Conclusion:**

MicroRNAs and metabolomics shows promising potential as new diagnostic methods due to their high diagnostic value compared with traditional biomarkers.

## INTRODUCTION

Hepatocellular carcinoma (HCC) has the sixth highest cancer morbidity and the second highest mortality rate worldwide. The ratio of deaths to new cases for liver cancer is 0.95 each year, while colorectal cancer, which has a better prognosis, is 0.51 [1]. Currently, the diagnosis of HCC relies on biopsy, imaging reports (ultrasound B, CT or MRI) and alpha feto-protein (AFP), according to the American Association for the Study of Liver Diseases (AASLD) Practice Guidelines. However, the sensitivity and specificity of AFP is barely satisfactory [2], necessitating the discovery of circulating biomarkers with a higher diagnostic value. After screening a host of novel biomarkers, including DNAs, RNAs, proteins and low-molecular-weight metabolites [2, 3], we selected two methodology: the detection of serum microRNAs and metabolomics based on gas chromatography/mass spectrometry (GC/MS), validated their diagnostic value and established appropriate models.

MicroRNAs are small, endogenous, non-coding RNAs that can regulate the expression of genes at the post-transcriptional level [4]. MicroRNAs can be released into peripheral blood when liver cell damage occurs [5]. During the past ten years, decades of studies have shown that diverse microRNAs possess great potential for the diagnosis of HCC. Therefore, it is essential to summarize the diagnostic efficiencies of these microRNAs via a systematic review. It is a pity that there are deficiencies in the published systematic reviews and meta-analyses. Some of these studies reviewed only one microRNA [6–9], while others conducted a meta-analysis including the whole diagnostic tests, but lacked the information on each microRNA [10–13]. We tried to overcome these disadvantages by selecting seven microRNAs with high Youden indexes and area under the curve (AUC) values of the receiver operating curve (ROC) to develop a diagnostic panel.

Metabolomics is defined as the quantitative measurement of all small molecule metabolites in an organism at a specified time under specific environmental conditions [14]. Rapid development in metabolomics has made it a promising technology in disease diagnosis and biomarker generation [15]. Compared with other metabolomic techniques, such as nuclear magnetic resonance (NMR) and liquid chromatography/mass spectrometry (LC/MS), GC/MS has a more robust result and is widely used in metabolite identification based on its high sensitivity, peak resolution, and reproducibility [16]. Several studies have reported the diagnostic value of metabolomics in HCC [17]. We further validated the diagnostic accuracy of GC/MS analysis and compared the most frequently used statistical methods.

## RESULTS

### Study selection and literature characteristics

The initial search from the databases and other sources returned a total of 590 articles, of which, 226 were from PubMed, 271 were from Embase, and 93 were from the Chinese Biomedical Literature Database (CBM). After removing 131 duplicates, 372 irrelevant studies and five articles that failed to provide enough diagnostic information, 82 published studies were enrolled into this systematic review (Supplementary Table 1). A total of 6035 HCC patients and 8181 healthy control (HCs) were included. The characteristics of the 82 studies are displayed in Supplementary Table 2.

### Diagnostic value of microRNAs in the literature

92 microRNAs were mentioned in the included articles, of which, 65 were studied in a single article. We conducted the meta-analyses to represent the diagnostic accuracy of the other 27 microRNAs. The details of their corresponding diagnostic value are shown in Table 1.

**Table 1:** Characteristics of the microRNAs mentioned in the literature

MicroRNA	Expression	HCC sample size	Control sample size	Sensitivity (%)	Specificity (%)	AUC	Number of included articles
miR-223	Upregulated & Downregulated	253	235	93.3	84.2	0.950	4
miR-146a	Upregulated	112	167	96.4	67.1	0.940	1
miR-30c	Downregulated	55	110	81.8	71.8	0.932	1
miR-152	Downregulated	112	145	88.6	87.8	0.930	2
miR-186	Upregulated	55	110	78.2	63.6	0.927	1
miR-595	Upregulated	87	31	81.7	93.2	0.920	1
miR-130b	Upregulated	57	59	87.7	81.4	0.913	1
miR-130a	Upregulated	112	42	96.4	78.4	0.910	1
miR-338	Upregulated & Downregulated	156	257	87.1	86.8	0.910	3
miR-34a	Upregulated	112	167	98.6	73.3	0.910	1
miR-182	Upregulated	203	315	83.1	86.5	0.910	3
miR-30e	Downregulated	39	31	92.3	71.0	0.910	1
miR-96	Upregulated	60	180	83.3	80.8	0.902	1
miR-224	Upregulated	347	545	87.6	82.5	0.900	4
miR-7	Downregulated	30	60	76.7	85.0	0.898	1
miR-145	Upregulated & Downregulated	332	483	96.3	75.8	0.890	2
miR-331-3p	Upregulated	103	95	79.6	89.5	0.890	1
miR-21	Upregulated & Downregulated	943	1176	84.5	80.6	0.890	13
miR-125b	Downregulated	443	602	84.3	80.8	0.890	4
miR-214-5p	Downregulated	224	334	81.8	83.3	0.890	1
miR-16-2	Upregulated & Downregulated	233	158	84.6	79.9	0.890	3
miR-3126-5p	Downregulated	115	40	87.0	78.4	0.881	1
miR-301	Upregulated	42	38	88.1	70.3	0.880	1
miR-19a	Downregulated	112	167	81.5	82.1	0.870	1
miR-150	Downregulated	120	230	80.8	80.0	0.870	1
miR-143	Upregulated & Downregulated	401	428	76.4	81.3	0.860	4
miR-29b	Downregulated	87	96	75.9	89.5	0.855	1
miR-4651	Upregulated	279	662	78.1	92.1	0.850	1
miR-106b	Upregulated & Downregulated	206	595	76.7	80.0	0.850	5
miR-574-3p	Upregulated	90	90	78.9	77.8	0.850	1
miR-26b	Downregulated	50	50	86.0	90.0	0.843	1
miR-1269	Upregulated	224	334	90.7	69.7	0.840	1
miR-939	Upregulated	87	31	85.8	73.7	0.840	1
miR-122 (miR-122a)	Upregulated & Downregulated	683	682	77.1	77.4	0.840	8
miR-10b	Upregulated	27	81	77.8	76.5	0.840	1
miR-101	Upregulated & Downregulated	156	333	76.7	75.7	0.820	3
miR-519d	Upregulated	87	31	72.4	78.4	0.820	1
miR-215	Upregulated	95	127	80.0	91.0	0.816	1
miR-27b-3p	Upregulated	91	91	63.0	89.0	0.812	1
miR-27a	Downregulated	90	60	86.7	65.0	0.811	1
miR-138b	Downregulated	224	334	88.3	69.1	0.810	1
miR-18a	Upregulated	101	90	81.8	73.1	0.810	1
miR-192	Upregulated & Downregulated	492	722	77.6	74.6	0.810	5
miR-203	Upregulated & Downregulated	107	158	72.5	76.5	0.810	2
miR-221	Upregulated & Downregulated	192	328	77.6	70.8	0.810	4
miR-4281	Upregulated	45	45	84.4	73.3	0.805	1
miR-15b	Upregulated	133	176	85.2	58.3	0.800	3
miR-4429	Upregulated	69	87	75.0	70.0	0.798	1
miR-764	Upregulated	37	60	74.5	77.0	0.791	1
miR-29a	Upregulated & Downregulated	138	209	77.3	82.3	0.790	2
miR-183	Upregulated	95	111	68.5	75.3	0.790	2
miR-185-5p	Upregulated	67	82	91.0	39.0	0.788	1
miR-6086	Upregulated	45	45	71.1	71.1	0.782	1
miR-195	Downregulated	112	167	83.4	65.9	0.780	1
miR-494	Upregulated	224	334	76.8	65.9	0.780	1
miR-296	Downregulated	112	167	76.8	64.6	0.780	1
miR-451a	Downregulated	66	40	95.0	81.8	0.770	1
miR-20a-5p	Upregulated	67	82	86.6	57.3	0.770	1
miR-92a-3p	Upregulated	182	122	69.0	73.6	0.770	2
miR-205	Downregulated	98	175	89.9	66.9	0.760	2
miR-483-5p	Upregulated	161	190	74.8	79.1	0.760	2
miR-199 (miR-199a)	Downregulated	266	455	67.6	80.8	0.760	4
miR-181a	Downregulated	27	81	74.2	67.7	0.760	1
miR-26a	Upregulated & Downregulated	277	367	68.6	72.8	0.760	3
miR-141	Upregulated & Downregulated	157	259	60.3	78.8	0.760	2
miR-335	Downregulated	50	40	78.0	70.0	0.750	1
miR-505	Upregulated	108	149	90.7	56.4	0.736	1
miR-218	Downregulated	156	162	66.7	69.1	0.734	1
miR-133a	Upregulated	108	149	64.8	81.9	0.733	1
miR-375	Downregulated	302	490	90.4	68.7	0.730	2
miR-107	Upregulated	115	40	75.4	62.5	0.730	1
miR-202	Downregulated	70	30	91.6	65.0	0.723	1
miR-132-3p	Upregulated	67	82	91.0	36.6	0.722	1
miR-25-3p	Upregulated	67	82	55.3	79.3	0.718	1
miR-148b	Downregulated	76	117	48.0	80.3	0.710	1
miR-29c	Upregulated	108	149	77.8	63.1	0.704	1
miR-129	Downregulated	23	55	81.8	69.7	0.700	1
miR-30a-5p	Upregulated	67	82	64.2	68.3	0.681	1
miR-155	Downregulated	23	55	86.2	62.3	0.680	1
miR-148a	Downregulated	76	117	55.5	85.6	0.680	1
miR-320a	Upregulated	67	82	38.8	87.8	0.678	1
miR-200a	Downregulated	22	37	56.9	92.6	0.670	1
miR-206	Upregulated	135	222	85.2	52.3	0.665	1
miR-500a	Upregulated	112	141	47.2	81.9	0.660	1
miR-324-3p	Upregulated	67	82	74.6	50.0	0.656	1
miR-24-3p	Upregulated	72	31	57.9	79.5	0.636	1
miR-let-7b	Upregulated	120	30	82.5	46.7	0.633	1
miR-433-5p	Upregulated	135	222	83.0	39.2	0.607	1
miR-126	Upregulated & Downregulated	82	103	83.7	51.8	0.600	2
miR-142-3p	Upregulated	59	48	32.0	91.0	0.553	1
miR-222	Upregulated	60	40	55.0	50.0	0.541	1
miR-1228-5p	Upregulated	135	222	66.7	43.7	0.534	1

The upregulated or downregulated expression trend in the HCC patients versus the control group. The data on the sensitivity, specificity and AUC were obtained via the meta-analysis when the number of included articles was more than one.

Abbreviations: HCC, hepatocellular carcinoma; AUC, area under the curve.

### Publication bias

**Figure 1:**
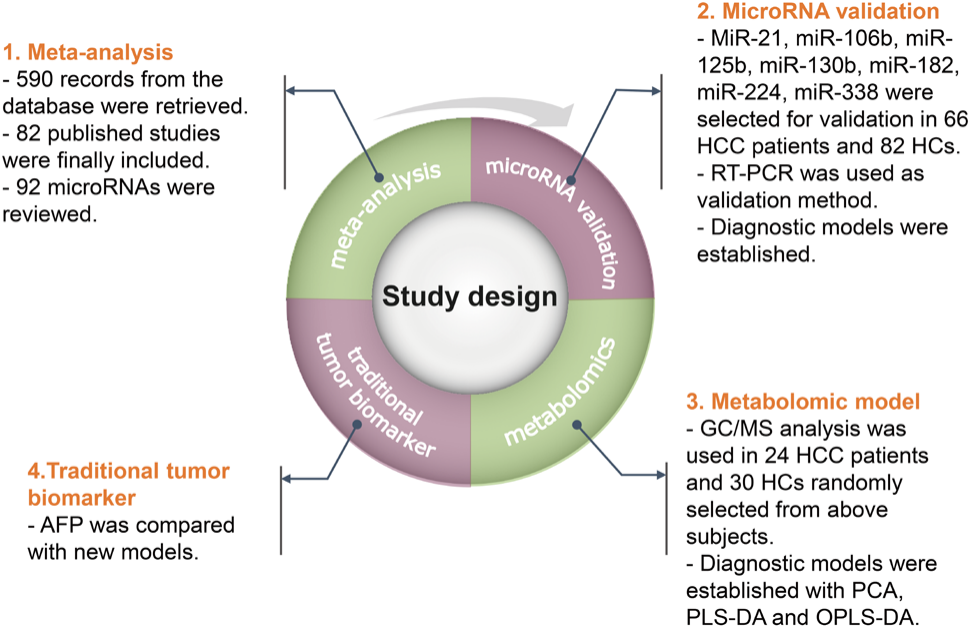
Deeks’ funnel plot for the assessment of publication bias

A Deeks’ funnel plot was used to evaluate publication bias (Figure 1), and the *P* values of Deeks’ tests was 0.08, which indicated no significant publication bias was observed in this analysis.

### Study population

The clinical and pathological characteristics of the study participants are presented in Table 2. The age and gender ratio were significantly different between HCC patients and HCs, thus, a covariance analyses were conducted. The results suggested that age and gender ratio was unrelated to the expression of the microRNAs, scores of the components and concentration of AFP.

**Table 2:** Clinical characteristics of the study population

Variable	Patients (n=66)	Control subjects (n=82)
Age (year)	57.9 ± 9.9	34.7 ± 7.3
Gender		
Male	53	49
Female	13	33
ALT (U/L)	34.0 ± 29.6	20.5 ± 13.2
<45	56	78
≥45	10	4
Tumor size (cm)	5.21 ± 3.81	
<5	39	
≥5	27	
TNM stage		
I	25	
II	16	
III	19	
IV	6	
Histological grade		
II	22	
III	10	
II∼III	15	
Unknown	19	
Etiology		
HBV	52	
HCV	2	
Fatty	4	
Unknown	8	
Liver cirrhosis		
Yes	52	
No	14	

Abbreviation: ALT, alanine aminotransferase; TNM, tumor-node-metastasis; HBV, hepatitis B virus; HCV, hepatitis C virus.

### Expression of microRNAs

**Figure 2:**
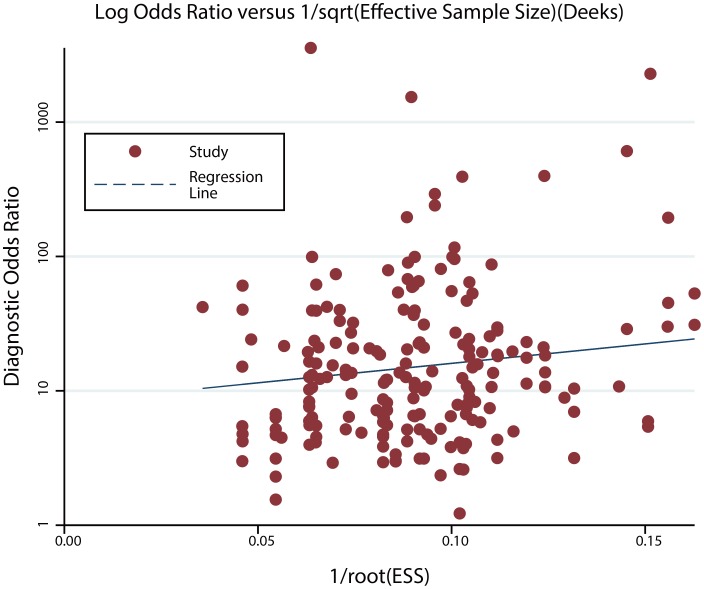
Box plots for the expression of the seven microRNAs The *P* values of miR-21, miR-106b, miR-125b, miR-130b, miR-182, miR-224 and miR-338 were < 0.001, 0.008, < 0.001, 0.224, 0.028, <0.001 and 0.070, respectively. The lines within the boxes represent the median values, and the edges of the boxes demonstrate the interquartile ranges. The lines outside the boxes demonstrate the 95% ranges. The points outside the boxes represent the values beyond the 95% ranges. Abbreviations: HCC, hepatocellular carcinoma; HC, healthy control.

MiR-21, miR-106b, miR-125b, miR-130b, miR-182, miR-224 and miR-338 were selected through the systematic review. The results of the quantitative reverse-transcription polymerase chain reaction (qRT-PCR) indicated that the serum levels of miR-21, miR-106b and miR-125b in the HCC patients were significantly higher than those in HCs, while those of miR-182 and miR-224 were significantly lower. As for miR-130b and miR-338, no significant difference was observed between HCC patients and HCs (Supplementary Table 3 and Figure 2). The expression of all of the seven microRNAs had no significant differences among four TNM stages (Kruskal-Wallis test, P > 0.05).

### Diagnostic models established using microRNAs

Table 3 presents the cut-off value, sensitivity, specificity, Youden index and AUC of each microRNA and their combinations. The combination of miR-21, miR-106b and miR-224 had the highest AUC value at 0.950, with a sensitivity of 80.3% and a specificity of 92.7%. The cut-off value of the model was -8.99, according to the formula miR-21 × 2.271 + miR-106b × 1.647 + miR-224 × (-3.306).

**Table 3:** Diagnostic value of five single microRNAs and their combinations

MicroRNA(s)	Sensitivity (%)	Specificity (%)	AUC (95% CI)	Cut-off value	Youden index
miR-21	98.5	59.8	0.872 (0.818, 0.925)	2.700	0.582
miR-106b	56.1	69.5	0.628 (0.538, 0.718)	3.008	0.256
miR-125b	77.3	58.5	0.693 (0.608, 0.779)	1.910	0.358
miR-182	56.1	65.2	0.605 (0.513, 0.697)	2.870	0.212
miR-224	43.9	90.9	0.715 (0.631, 0.799)	6.995	0.348
miR-21+miR-106b	98.5	61.0	0.872 (0.818, 0.926)	4.985	0.595
miR-21+miR-125b	90.9	64.6	0.873 (0.819, 0.926)	5.384	0.555
miR-21+miR-182	72.7	90.2	0.905 (0.860, 0.950)	4.917	0.630
miR-21+miR-224	75.8	95.1	0.924 (0.883, 0.964)	-4.642	0.709
miR-106b+miR-125b	72.7	59.8	0.684 (0.598, 0.770)	1.370	0.325
miR-106b+miR-182	72.7	70.7	0.751 (0.674, 0.829)	0.809	0.435
miR-106b+miR-224	68.2	91.5	0.872 (0.817, 0.927)	-11.789	0.596
miR-125b+miR-182	86.4	65.9	0.777 (0.701, 0.853)	-0.546	0.522
miR-125b+miR-224	71.2	85.4	0.846 (0.785, 0.908)	-9.422	0.566
miR-182+miR-224	80.3	58.5	0.741 (0.660, 0.821)	-9.163	0.388
miR-21+miR-106b+miR-125b	95.5	59.8	0.873 (0.819, 0.926)	4.859	0.552
miR-21+miR-106b+miR-182	95.5	74.4	0.914 (0.872, 0.956)	4.858	0.698
miR-21+miR-106b+miR-224	80.3	92.7	0.950 (0.920, 0.980)	-8.986	0.730
miR-21+miR-125b+miR-182	90.9	75.6	0.913 (0.870, 0.955)	3.811	0.665
miR-21+miR-125b+miR-224	81.8	92.7	0.938 (0.902, 0.974)	-8.325	0.745
miR-21+miR-182+miR-224	75.8	95.1	0.924 (0.883, 0.964)	-5.105	0.709
miR-106b+miR-125b+miR-182	90.9	58.5	0.789 (0.716, 0.862)	0.168	0.494
miR-106b+miR-125b+miR-224	89.4	78.0	0.897 (0.848, 0.946)	-14.671	0.674
miR-106b+miR-182+miR-224	74.2	85.4	0.873 (0.818, 0.928)	-13.746	0.596
miR-125b+miR-182+miR-224	74.2	81.7	0.854 (0.794, 0.915)	-12.424	0.559
miR-21+miR-106b+miR-125b+miR-182	95.5	74.4	0.915 (0.873, 0.957)	4.703	0.698
miR-21+miR-106b+miR-125b+miR-224	80.3	96.3	0.953 (0.923, 0.982)	-10.320	0.766
miR-21+miR-106b+miR-182+miR-224	80.3	92.7	0.950 (0.920, 0.980)	-8.553	0.730
miR-21+miR-125b+miR-182+miR-224	81.8	92.7	0.936 (0.900, 0.973)	-10.653	0.745
miR-106b+miR-125b+miR-182+miR-224	81.8	80.5	0.896 (0.846, 0.946)	-16.120	0.623
miR-21+miR-106b+miR-125b+miR-182+miR-224	78.8	96.3	0.952 (0.923, 0.981)	-10.813	0.751

The bold font indicates that the P value of each microRNA in the combination was less than 0.05 in the logistic regression.

Abbreviation: AUC, area under the curve; CI, confidence interval.

### Discrepant metabolites and total ion chromatogram

A total of 1118 features were extracted in this experiment. Seventeen significantly different metabolites are presented in Supplementary Table 4. The retention time (RT) in the total ion chromatograms was stable with no drift in all of the peaks, which indicated that the results were reliable.

### Diagnostic models established using metabolomics

First, we performed the multivariate statistical analyses in all 1118 metabolites. In the principal component analysis (PCA) model, we extracted ten principal components, seven of whose eigenvalue were more than 1.0. We calculated the diagnostic parameters when fitting into one to ten principal components (Table 4). As shown, the AUC was higher as the number of the principal components fitted into the model were increased. We extracted one component in partial least squares-discriminate analysis (PLS-DA) and orthogonal partial least squares-discriminant analysis (OPLS-DA) model, respectively, and the AUC reached 0.89 and 1.0.

**Table 4:** Diagnostic value of the gas chromatography/mass spectrometry analysis with multivariate statistical analysis methods

Source of components	Statistical method	Number of components	Sensitivity (%)	Specificity (%)	AUC (95% CI)	Youden index	Cumulative variance
All metabolites	PCA	10	100.0	100.0	1.000 (1.000, 1.000)	1.000	0.783
		9	100.0	100.0	1.000 (1.000, 1.000)	1.000	0.777
		8	75.0	96.7	0.924 (0.857, 0.990)	0.717	0.767
		7	75.0	96.7	0.921 (0.852, 0.990)	0.717	0.745
		6	58.3	100.0	0.876 (0.788, 0.965)	0.583	0.725
		5	62.5	100.0	0.883 (0.797, 0.970)	0.625	0.694
		4	95.8	60.0	0.857 (0.761, 0.953)	0.558	0.635
		3	95.8	60.0	0.854 (0.757, 0.951)	0.558	0.589
		2	95.8	60.0	0.854 (0.757, 0.951)	0.558	0.543
		1	70.8	50.0	0.564 (0.409, 0.719)	0.208	0.329
	PLS-DA	1	83.3	76.7	0.894 (0.815, 0.974)	0.600	0.341
	OPLS-DA	1	100.0	100.0	1.000 (1.000, 1.000)	1.000	0.704
Significantly different metabolites	PCA	5	100.0	100.0	1.000 (1.000, 1.000)	1.000	0.744
		4	100.0	100.0	1.000 (1.000, 1.000)	1.000	0.734
		3	95.8	96.7	0.994 (0.983, 1.000)	0.925	0.713
		2	95.8	100.0	0.994 (0.982, 1.000)	0.958	0.673
		1	100.0	93.3	0.989 (0.970, 1.000)	0.933	0.413
	PLS-DA	1	100.0	93.3	0.996 (0.986, 1.000)	0.933	0.628
	OPLS-DA	1	100.0	93.3	0.996 (0.986, 1.000)	0.933	0.628

Abbreviations: AUC, area under the curve; CI, confidence interval; PCA, principal component analysis; PLS-DA, partial least squares-discriminate analysis; OPLS-DA, orthogonal partial least squares-discriminant analysis.

When the seventeen significantly different metabolites were used to diagnose HCC, the AUC reached 1.0. Further multivariate statistical analyses also displayed promising results. In the PCA model, we extracted five principal components, three of whose eigenvalue was more than 1.0. The AUC reached 1.0 when more than four principal components were included. Only one component was extracted in both of PLS-DA and OPLS-DA model, and the AUC both reached 0.996.

**Figure 3:**
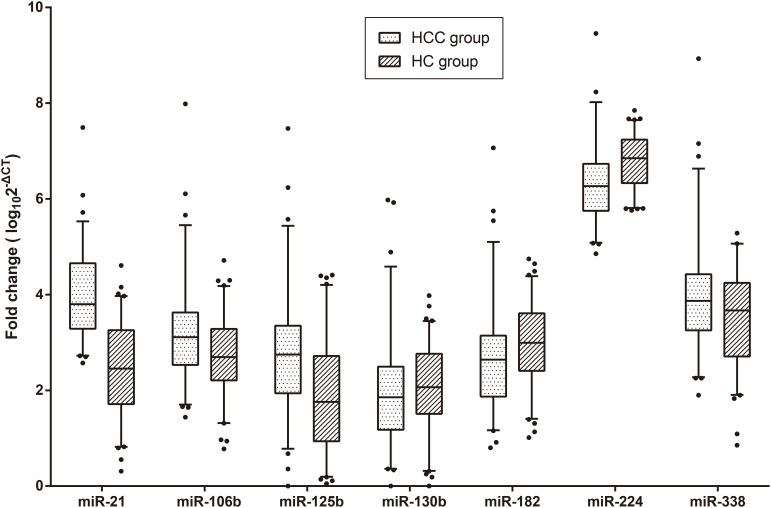
Score plots of the GC/MS analysis in the hepatocellular carcinoma patients and healthy controls ○ represents the hepatocellular carcinoma group. ▲ represents the healthy control group. The scatter plots of the principal component analysis (PCA) with two principal components for all metabolites (1A) and significantly different metabolites (1B). The line within the plot represents the optimal cut-off line. The strip charts of the partial least squares-discriminate analysis (PLS-DA) with the only component for all metabolites (2A) and significantly different metabolites (2B). The strip charts of the orthogonal partial least squares-discriminant analysis (OPLS-DA) with the only component for all metabolites (3A) and significantly different metabolites (3B).

More diagnostic information regarding the multivariate statistical analyses is shown in Table 4 and Figure 3.

### Diagnostic value of traditional tumor biomarkers

The AFP concentration was significantly different between HCC patients and HCs (Mann-Whitney *U*-test, *P* < 0.001). The median concentrations in the patients and HCs were 42.2 (range, 1.2 - > 60500) and 3.6 (range, 0.9 – 10.3) μg/L, respectively. The AUC of AFP was 0.755 (95% CI, 0.666 - 0.843; sensitivity = 59.1%, specificity = 100.0%) when the cut-off value was 12.3 μg/L. When the cut-off value was 20 μg/L, which is the upper bound of 95% of healthy individuals, the sensitivity was 54.5%, and the specificity was still 100.0%.

**Figure 4:**
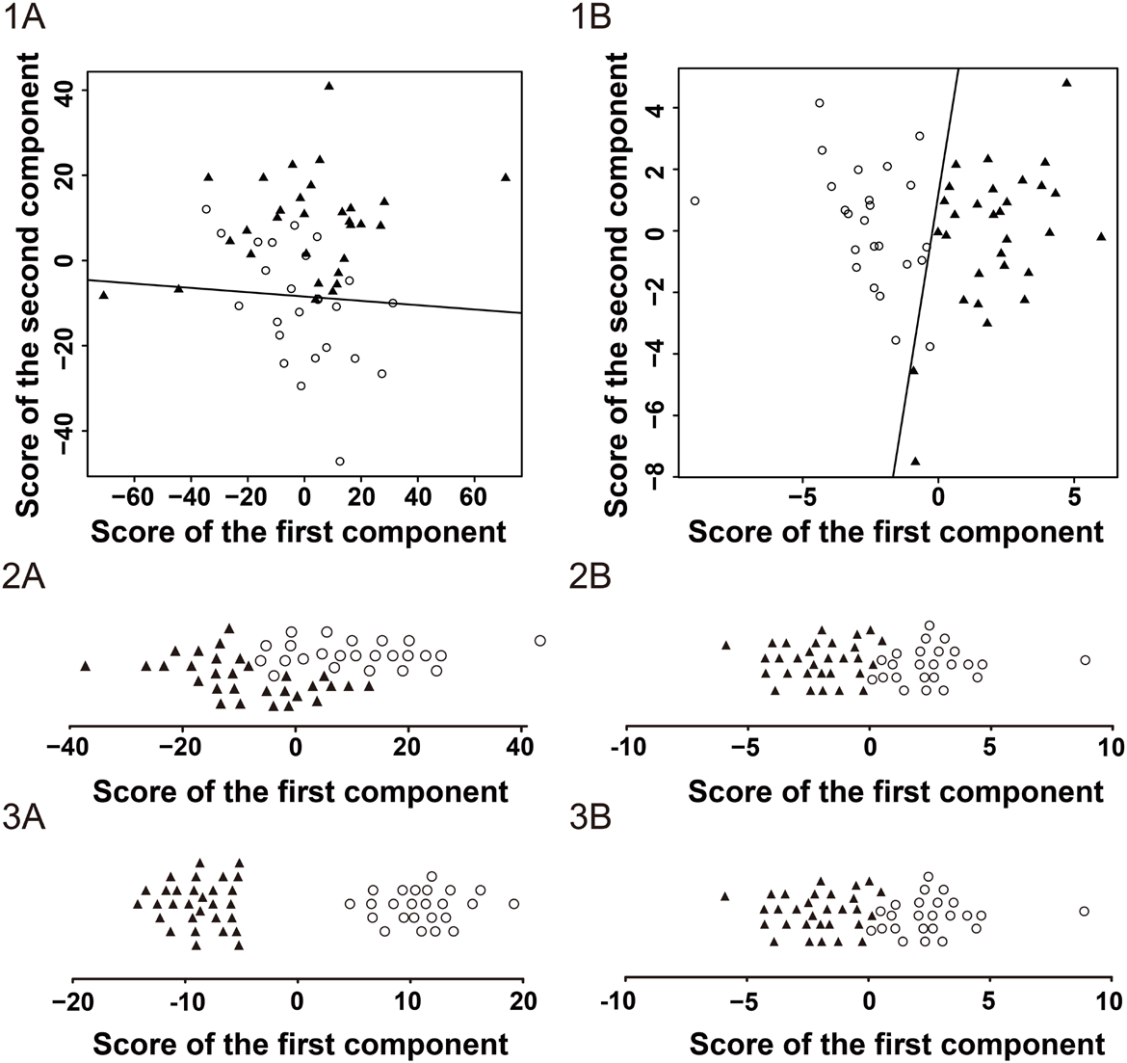
Receiver operating characteristic (ROC) curve ROC curve of the combination of miR-21, miR-106b and miR-224, GC/MS analysis with three statistical methods for all metabolites and AFP for discriminating hepatocellular carcinoma patients from control subjects. The curve of PCA model was performed when including two principal components. Abbreviations: PCA, principal component analysis; PLS-DA, partial least squares-discriminate analysis; OPLS-DA, orthogonal partial least squares-discriminant analysis; AFP, alpha feto-protein.

The ROC curves of AFP, metabolomics and the combination of microRNAs are displayed in Figure 4.

## DISCUSSION

Early diagnosis and treatment of HCC can improve patient survival is a well-established consensus. Thus, looking for new biomarkers is in the ascendant. Novel diagnostic biomarkers almost belong to gene mutations, single nucleotide polymorphisms (SNP), epigenetics, mRNAs, non-coding RNAs and proteins including GPC3, GP73, DKK1 [18, 19]. Screening via proteomics or metabolomics is also a feasible way to discover new biomarkers. After investigating the diagnostic efficiencies and limitations of biomarkers, we selected serum microRNAs and GC/MS to validate their diagnostic value and establish appropriate models.

Among the thousands of microRNAs that have been discovered, many have been testified for their diagnostic value in HCC [10]. A general research routine is through screening microRNA microarray in a small sample size, then validating the results via qRT-PCR in a larger sample size. We reviewed the diagnostic value of each microRNA. Meta-analyses made the statistical power increase through the expansion of the included articles and sample sizes.

Based on the result of systematic review, we selected seven microRNAs with high AUC values or Youden indexes that were included in various articles. MiR-21, miR-106b, miR-125b, miR-182 and miR-224 had significantly different expression in HCC patients versus HCs. AUC higher than 0.7, miR-21 and miR-224 had potential to become independent diagnostic biomarkers of HCC. The combination of microRNAs further raised the diagnostic value and the combination of miR-21, miR-106 and miR-224 allowed the AUC to exceed 0.950. With miR-21, miR-106b, miR-125b, miR-182 and miR-224 combined, the AUC was 0.952. However, there was no significant difference between the above two combinations.

As circulating diagnostic biomarkers, microRNAs have advantages and disadvantages. Different from mRNAs, microRNAs are stable at room temperature and remains so after repeated freeze-thawing [20]. In addition, compared with liver puncture, blood examination is non-invasive. Nevertheless, the choice of internal/external reference RNA, the dosage of reagents and the operating process lacks standardization, therefore the cut-off value cannot be unified, and even the variation trend of the expression for some microRNAs are distinct. On the other hand, the etiology, such as hepatitis B virus or hepatitis C virus, may affect the expression of microRNAs.

As expected, the diagnostic efficiency of metabolomics is satisfactory, whether all detected metabolites or significantly different metabolites were included. As shown in Table 4 , when a PCA, PLS-DA or OPLS-DA model includes the same number of components, the OPLS-DA model had the highest AUC, and the PCA model ranks last. This conclusion can be explained from a mathematics perspective. PCA is non-supervisory, while PLS-DA and OPLS-DA are supervisory analysis methods. Based on PLS, OPLS further separates the orthogonal variables by an orthogonal signal correction and expands the differences between the two data matrices [21, 22]. Although the diagnostic value of the PCA model was not superior to that of the PLD-DA and OPLS-DA model when including the same number of components, the PCA can extract more principal components to increase the AUC.

The advantage of serum GC/MS analysis are high diagnostic value and non-invasive examination process. The statistical models, which are established by PCA, PLA-DA and OPLS-DA, are stable when the variables are numerous and the observations are little. Nevertheless, same as detecting the expression of microRNAs, the pretreatment process is not standardized, including the choice of the derivatization reagents and internal standard, the time of each step and the operating order.

In terms of the price, new biomarker detections are more expensive than traditional AFP, which costs only 5.2 dollars in China. Each sample detection for three microRNAs and the metabolic spectra costs approximately 20 and 72.5 dollars, respectively. Moreover, an abdominal enhancement CT and enhancement MRI are priced around 100 and 135 dollars, respectively. A liver puncture costs 44 dollars, excluding test-related room and nursing care charges.

In conclusion, the diagnostic value of the new models are higher than that of the traditional biomarker, AFP, without doubt. We suggest GC/MS analysis and a combination of microRNAs applied to the diagnosis of HCC, especially after the position diagnosis is made via imaging examination.

## MATERIALS AND METHODS

### Study design

First, an electronic search of PubMed, Embase and the CBM databases was performed to identify relevant articles published up to July 6, 2017. The search strategy was (miRNA OR microRNA OR miR) AND (“liver neoplasms”[Mesh] OR “hepatocellular carcinoma” OR “liver cancer”) AND (blood OR serum OR plasma OR circulating) AND (diagnosis OR diagnostic OR diagnose). In addition, we examined the reference lists in identified articles to included additional relevant studies. No language restrictions were imposed.

Secondly, we chose microRNAs with high AUC values and is included in numerous studies to establish a diagnostic model. The serum specimens from 66 HCC patients and 82 HCs were collected to detect the expression of microRNAs through qRT-PCR.

Next, we randomly selected 24 patients and 30 HCs from the cohort mentioned above and profiled their metabolomic signatures via GC/MS analysis.

**Figure 5:**
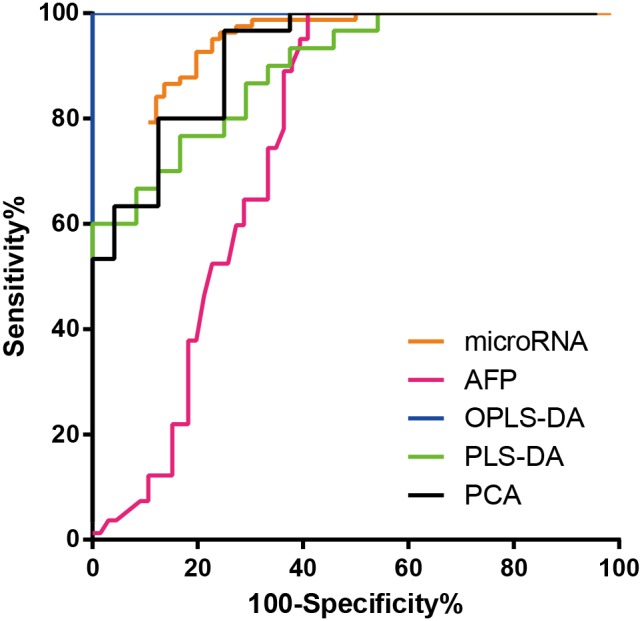
Study design Abbreviations: HCC, hepatocellular carcinoma; HC, healthy control; RT-PCR, reverse-transcription polymerase chain reaction; GC/MS, gas chromatography/mass spectrometry; PCA, principal component analysis; PLS-DA, partial least squares-discriminate analysis; OPLS-DA, orthogonal partial least squares-discriminant analysis; AFP, alpha feto-protein.

Finally, we detected the serum concentration of the traditional tumor biomarker, AFP. The diagnostic efficiency was calculated and compared to the new models. The flow-process diagram for the study is shown in Figure 5.

### Inclusion and exclusion criteria of the literature

The inclusion criteria for the systematic review were as follows: (1) studies regarding microRNAs comparing HCC patients with HCs; (2) studies that employed blood specimens, including serum and plasma; and (3) qRT- PCR techniques. The exclusion criteria included: (1) failure to provide sufficient diagnostic information; (2) duplicate data from identical authorities; and (3) cell or animal studies, reviews and letters.

### Data extraction

Two reviewers were independently responsible for study selection and data extraction. Data were retrieved from all included studies: (1) basic characteristics of the studies, including the first author, year of publication, country, ethnicity, sample size, mean age, gender, type of specimens, target microRNAs, and reference control; and (2) diagnostic parameters of the microRNAs, including expression variation, sensitivity, specificity, and AUC.

### Patients and specimens

In this study, we included patients and HCs from Zhongshan Hospital, Fudan University between May 2015 and July 2015. The HCC patients were all definitively diagnosed in accordance with the AASLD Practice Guidelines. The patients were excluded if they had history of other malignant tumors or had received surgical operation, interventional therapy, radiotherapy or chemotherapy. Healthy individuals were identified by clinical manifestations, histories of illness and normal liver function. The serum samples were centrifuged for 10 min at 820 g and 4°C to remove cell debris, and the supernatants were immediately stored at −80°C until analysis. The concentration of serum AFP was measured via an electro-chemiluminescence immunoassay.

The protocol was approved by the Ethics Committee of Zhongshan Hospital of Fudan University, Shanghai. All participants provided a written informed consent.

### RNA extraction and reverse transcription

2 μl of 25 fmol cel-miR-39 (Tiangen, Beijing, China) was added to 200 μl of serum samples as external reference. Total RNA was isolated simultaneously using the miRcute microRNA Isolation Kit (Tiangen, Beijing, China) abiding by the manufacturer’s protocol [23]. The optical density of the extracted total RNA was determined at 260 and 280 nm on a NanoDrop spectrophotometer (NanoDrop, Wilmington, DE, USA) to assess for concentrations and purities.

The extracted microRNA was polyadenylated with poly (A) polymerase in a 20-μl volume, and 6 μl of the poly (A) reaction solution was reversely transcribed to cDNA in another 20 μl with miRcute microRNA The First-strand cDNA Synthesis Kit (Tiangen, Beijing, China) according to the manufacturer’s protocol. All procedures were carried out in triplicates to remove outliers.

### Quantitative real-time PCR

The qPCR reaction was conducted with the miRcute microRNA qPCR Detection Kit (Tiangen, Beijing, China) on ABI PRISM 7500 Sequence Detection System (Applied Biosystems, Foster City, CA, USA). Each 20-μl qPCR reaction solution contained cDNA, 2× miRcute microRNA premix (with SYBR and ROX), the manufacturer-provided universal reverse primer, and a microRNA-specific forward primer (Tiangen, Beijing, China). The real-time PCR cycling conditions: 94°C for 2 min, 45 cycles at 94°C for 20 s, annealing at 60°C for 34 s, and extension at 72°C for 30 s. At the end of the real-time PCR reaction, a melting curve analysis was accomplished to ensure specific amplification of the expected PCR product.

The relative expression of the microRNAs was calculated from the equation log_10_ (2^−ΔCt^) with cel-miR-39. The ΔCT was calculated by subtracting the CT values of the cel-miR-39 from those of the microRNAs of interest [23].

### Specimen processing for metabolomics

200 μl of the serum samples were transferred into glass centrifuge tubes for GC/MS analysis. 200 μl of 2-chloro-phenylalanine (0.3 g/L) served as internal standard. 600 μl of methanol was added into each sample. The mixture was vortexed for 30 s, followed by incubation at -20°C for 10 min. The samples were then centrifuged for at 12000 × g and 4°C for 15 min. 800 μl of the supernatant was collected individually from each sample into an ampoule bottle and evaporated to dryness under a stream of nitrogen gas at 50°C for approximately 30 min. 200 μl of a methoxyamine pyridine solution (15 g/L) was subsequently added into the ampoule bottle. The mixture was vortexed for 2 min and incubated at 37°C for 1 hour. Then, 200 μl of bis-(trimethylsilyl)-trifluoroacetamide (BSTFA) plus 1% trimethylchlorosilane (TMCS) was added, and the mixture was again vortexed for 2 min and incubated at 100°C for 30min. The methanol, 2-chloro-phenylalanine, methoxyamine and pyridine were obtained from Aladdin (Shanghai, China). BSTFA with 1% TMCS was purchased from Sigma-Aldrich (St. Louis, MO, USA). Each reaction sample was performed in duplicates.

### GC/MS analysis

The GC/MS analysis was performed on an Agilent 6980 GC system equipped with a fused-silica capillary column (internal diameter: 30 m × 0.25 mm) and a 0.25-μm HP-5MS stationary phase (Agilent, Shanghai, China). We used the same operational methods as our previous studies [24].

### Statistical analyses

The statistical analyses were carried out using R software 3.3.3 (R Foundation for Statistical Computing, Vienna, Austria), Stata 12.0 (StataCorp LP, College Station, TX, USA) and SIMCA-P 13.0 (Umetrics AB, Umea, Vasterbotten, Sweden). *P* values < 0.05 were considered statistically significant.

Meta-analyses were used to assess the accuracy of individual microRNAs for HCC diagnosis, based on its sensitivity, specificity and AUC of the summary receiver operator characteristic (SROC). Deeks’ funnel plot was selected to evaluated publication bias.

A power analysis was used to calculate the number of cases and HCs in the microRNA validation phase. A Mann-Whitney *U*-test was used to compare the expression of microRNAs and concentration of AFP in HCC patients and HCs. A Kruskal-Wallis test was used to calculate the relationship between the expression of microRNAs and TNM stage. The diagnostic efficiencies of the microRNAs were determined by assessing the sensitivity, specificity and the AUC. A stepwise logistic regression was used to include microRNAs into the diagnostic model.

The metabolomic data were normalized with “XCMS” package in R software and then stored in a two-dimensional matrix, including the RT, mass-to-charge ratio (MZ) and peak intensity. The metabolites were identified based on the National Institute of Standards and Technology (NIST) mass spectra library through RT and MZ [24]. Significantly different metabolites were screened via the variable importance in the projection (VIP) value of the OPLS-DA model (> 1) and the *P* value of *t*-test (≤ 0.001). Multivariate statistical analyses, including the PCA, PLS-DA and OPLS-DA, were carried out via SIMCA-P in all metabolites and significantly different metabolites, respectively. A logistic regression was used to investigate the better diagnostic models by combinations of the components when more than one component was extracted.

## SUPPLEMENTARY MATERIALS TABLES







## Abbreviations

AASLD: American Association for the Study of Liver Diseases
AFP: alpha feto-protein
AUC: area under the curve
BSTFA: bis-(trimethylsilyl)-trifluoroacetamide
CBM: Chinese Biomedical Literature Database
CT: cycle threshold or computed tomography
DKK1: dickkopf-related protein 1
GC/MS: gas chromatography/mass spectrometry
GP73: Golgi glycoprotein 73
GPC3: glypican 3
HC: healthy control
HCC: hepatocellular carcinoma
LC/MS: liquid chromatography/mass spectrometry
MRI: magnetic resonance imaging
MZ: mass-to-charge ratio
NIST: National Institute of Standards and Technology
NMR: nuclear magnetic resonance
OPLS-DA: orthogonal partial least squares-discriminant analysis
PCA: principal component analysis
PLS-DA: partial least squares-discriminate analysis
qRT-PCR: quantitative reverse-transcription polymerase chain reaction
ROC: receiver operating curve
RT: retention time
SNP: single nucleotide polymorphisms
SROC: summary receiver operator characteristic
TMCS: trimethylchlorosilane
VIP: variable importance in the projection
