# Supplementary material for: Serum microRNA signatures and metabolomics have high diagnostic value in hepatocellular carcinoma
Source: Oncotarget. 2017 Nov 1;8(65):108810–24. doi: 10.18632/oncotarget.22224 (PMC5752483; doi:10.18632/oncotarget.22224)
Supplement: Supplementary file 1 [file oncotarget-08-108810-s001.pdf]

Serum microRNA signatures and metabolomics have high diagnostic value in hepatocellular carcinoma

SUPPLEMENTARY MATERIALS

Supplementary Table 1: List of the included studies

See Supplementary File 1

Supplementary Table 2: Characteristics of the included studies

See Supplementary File 2

Supplementary Table 3: microRNA expressions and the results of the Mann-Whitney *U*-test

	Fold change		Z value	P value
	HCC group	Control group		
miR-21	3.80 (3.35, 4.64)	2.46 (1.72, 3.23)	-7.762	<0.001
miR-106	3.11 (2.55, 3.62)	2.69 (2.22, 3.27)	-2.669	0.008
miR-125	2.75 (1.94, 3.32)	1.76 (0.97, 2.70)	-4.035	<0.001
miR-130	1.85 (1.20, 2.44)	2.07 (1.52, 2.76)	1.215	0.224
miR-182	2.64 (1.89, 3.12)	3.00 (2.44, 3.60)	2.191	0.028
miR-224	6.27 (5.79, 6.72)	6.85 (6.34, 7.23)	4.486	<0.001
miR-338	3.87 (3.26, 4.39)	3.67 (2.75, 4.24)	-1.809	0.070

The fold change in HCC group and control group is expressed by the format “median (lower quartile, upper quartile)”.
Abbreviations: HCC, hepatocellular carcinoma.

Supplementary Table 4: Significantly different metabolites between the hepatocellular carcinoma patients and the healthy controls

Significantly different metabolites	VIP	MZ	RT	Fold change
(-)-Cholesterol	1.518	232.219	32.26	-0.826
Cyclopentasiloxane	1.198	72.100	7.04	-0.246
Silanol	1.433	89.862	9.27	-0.304
Glucose oxime	1.630	170.119	18.06	0.246
D-Glucose	2.053	432.299	18.11	0.404
Heptacosane	1.344	169.156	17.96	0.319
Silane	1.766	65.076	32.55	-0.515
4,7,10,13,16,19-Docosahexaenoic acid	1.845	351.389	25.65	-0.727
D-Mannose	2.036	310.208	18.16	0.408
D-Mannitol	1.628	481.381	18.38	0.430
D-Galactose	2.230	434.299	18.11	1.645
Undecane	1.863	56.097	5.72	-0.247
Octadecanoic acid	1.630	262.300	27.86	-0.483
Ethanamine	2.013	58.099	23.69	-0.894
Xylitol	1.220	156.097	15.19	0.662
Pentacosane	1.603	412.381	27.99	-0.346
Heptasiloxane	1.973	415.365	28.02	-0.508

Abbreviations: VIP, variable importance in the projection; MZ, mass-to-charge ratio; RT, retention time.
