# Supplementary material for: Serum microRNA signatures and metabolomics have high diagnostic value in hepatocellular carcinoma
Source: Oncotarget. 2017 Nov 1;8(65):108810–24. doi: 10.18632/oncotarget.22224 (PMC5752483; doi:10.18632/oncotarget.22224)
Supplement: Supplementary file 2 [file oncotarget-08-108810-s002.docx]

**Supplementary Table 1: List of the included studies**

| ID | Article information |
| --- | --- |
| 1 | Tarek M, Louka ML, Khairy E, Ali-Labib R, Zakaria ZD, Montasser IF. Role of microRNA-7 and selenoprotein P in hepatocellular carcinoma. Tumour Biol. 2017; 39:1393391292. |
| 2 | Jiang L, Cheng Q, Zhang BH, Zhang MZ. Circulating microRNAs as biomarkers in hepatocellular carcinoma screening: a validation set from China. Medicine (Baltimore). 2015; 94:e603. |
| 3 | Liu AM, Yao TJ, Wang W, Wong KF, Lee NP, Fan ST, Poon RT, Gao C, Luk JM. Circulating miR-15b and miR-130b in serum as potential markers for detecting hepatocellular carcinoma: a retrospective cohort study. BMJ Open. 2012; 2:e825. |
| 4 | Chen Y, Chen J, Liu Y, Li S, Huang P. Plasma miR-15b-5p, miR-338-5p, and miR-764 as Biomarkers for Hepatocellular Carcinoma. Med Sci Monit. 2015; 21:1864-71. |
| 5 | Chen Y, Huang P, Chen J, Liu YZ, Li SL, Wang ZY, Song D. Plasma circulating miR-338-5p, miR-21-5p and miR-15b-5p are potential biomarkers for screening hepatocellular carcinoma. Acta Acad Med Mil Tert. 2015;37:1720-6. |
| 6 | El-Abd NE, Fawzy NA, El-Sheikh SM, Soliman ME. Circulating miRNA-122, miRNA-199a, and miRNA-16 as Biomarkers for Early Detection of Hepatocellular Carcinoma in Egyptian Patients with Chronic Hepatitis C Virus Infection. Mol Diagn Ther. 2015; 19:213-20. |
| 7 | El-Tawdi AH, Matboli M, Shehata HH, Tash F, El-Khazragy N, Azazy A, Abdel-Rahman O. Evaluation of Circulatory RNA-Based Biomarker Panel in Hepatocellular Carcinoma. Mol Diagn Ther. 2016; 20:265-77. |
| 8 | Zhang Y, Li T, Qiu Y, Zhang T, Guo P, Ma X, Wei Q, Han L. Serum microRNA panel for early diagnosis of the onset of hepatocellular carcinoma. Medicine (Baltimore). 2017; 96:e5642. |
| 9 | Li L, Guo Z, Wang J, Mao Y, Gao Q. Serum miR-18a: a potential marker for hepatitis B virus-related hepatocellular carcinoma screening. Dig Dis Sci. 2012; 57:2910-6. |
| 10 | Motawi TK, Shaker OG, El-Maraghy SA, Senousy MA. Serum MicroRNAs as Potential Biomarkers for Early Diagnosis of Hepatitis C Virus-Related Hepatocellular Carcinoma in Egyptian Patients. PLoS One. 2015; 10:e137706. |
| 11 | Wen Y, Han J, Chen J, Dong J, Xia Y, Liu J, Jiang Y, Dai J, Lu J, Jin G, Han J, Wei Q, Shen H, et al. Plasma miRNAs as early biomarkers for detecting hepatocellular carcinoma. Int J Cancer. 2015; 137:1679-90. |
| 12 | Guo X, Lv X, Lv X, Ma Y, Chen L, Chen Y. Circulating miR-21 serves as a serum biomarker for hepatocellular carcinoma and correlated with distant metastasis. Oncotarget. 2017; 8:44050-8. |
| 13 | Zhuang C, Jiang W, Huang D, Xu L, Yang Q, Zheng L, Wang X, Hu L. Serum miR-21, miR-26a and miR-101 as potential biomarkers of hepatocellular carcinoma. Clin Res Hepatol Gastroenterol. 2016; 40:386-96. |
| 14 | Amr KS, Ezzat WM, Elhosary YA, Hegazy AE, Fahim HH, Kamel RR. The potential role of miRNAs 21 and 199-a in early diagnosis of hepatocellular carcinoma. Gene. 2016; 575:66-70. |
| 15 | Tomimaru Y, Eguchi H, Nagano H, Wada H, Kobayashi S, Marubashi S, Tanemura M, Tomokuni A, Takemasa I, Umeshita K, Kanto T, Doki Y, Mori M. Circulating microRNA-21 as a novel biomarker for hepatocellular carcinoma. J Hepatol. 2012; 56:167-75. |
| 16 | Xu J, Wu C, Che X, Wang L, Yu D, Zhang T, Huang L, Li H, Tan W, Wang C, Lin D. Circulating microRNAs, miR-21, miR-122, and miR-223, in patients with hepatocellular carcinoma or chronic hepatitis. Mol Carcinog. 2011; 50:136-42. |
| 17 | Chen DD, Wang HL, Huang SD, Zeng LM. The clinical value of detecting four kinds of microRNA for diagnosis of hepatocellular carcinoma. Anhui Med J. 2016;37:582-6. |
| 18 | Gao L, Liu HF, Ling MZ, Tian WQ, Zhao MJ, Zhao WX, Xu PS. Circulating microRNA-21 in hepatocellular carcinoma patients. Chin J Lab Med. 2015;484-6. |
| 19 | Qin ZH, Zhu XD, Huang YS. Circulating microRNA-21 as a novel biomarker for hepatocellular carcinoma. Sichuan Medical Journal. 2013;34:1423-5. |
| 20 | Wang P, Mo JG, Zhu J, Li ZY. Serum miR-21 and miR-4429 levels detected by droplet digital PCR for diagnosis of hepatocellular carcinoma. Zhejiang Medical Journal. 2017;39:170-2. |
| 21 | Chen XH, Wang YL, Zhang L, Yin D, Gao Y, Zhang ZX. Diagnostic value of serum miR-21 combined with ultrasound in hepatocellular carcinoma. Medical Journal of Communications. 2016;30:465-7. |
| 22 | Meng FL, Wang W, Jia WD. Diagnostic and prognostic significance of serum miR-24-3p in HBV-related hepatocellular carcinoma. Med Oncol. 2014; 31:177. |
| 23 | Zuo D, Chen L, Liu X, Wang X, Xi Q, Luo Y, Zhang N, Guo H. Combination of miR-125b and miR-27a enhances sensitivity and specificity of AFP-based diagnosis of hepatocellular carcinoma. Tumour Biol. 2016; 37:6539-49. |
| 24 | Tan Y, Ge G, Pan T, Wen D, Chen L, Yu X, Zhou X, Gan J. A serum microRNA panel as potential biomarkers for hepatocellular carcinoma related with hepatitis B virus. PLoS One. 2014; 9:e107986. |
| 25 | Chen HY, Jia HY, Wu XM. Expression and significance of microRNA-26b in peripheral blood of patients with hepatocellular carcinoma. Journal of Chinese Practical Diagnosis and Therapy. 2016;30:1215-6. |
| 26 | Zhu HT, Liu RB, Liang YY, Hasan A, Wang HY, Shao Q, Zhang ZC, Wang J, He CY, Wang F, Shao JY. Serum microRNA profiles as diagnostic biomarkers for HBV-positive hepatocellular carcinoma. Liver Int. 2017; 37:888-96. |
| 27 | Lin XJ, Chong Y, Guo ZW, Xie C, Yang XJ, Zhang Q, Li SP, Xiong Y, Yuan Y, Min J, Jia WH, Jie Y, Chen MS, et al. A serum microRNA classifier for early detection of hepatocellular carcinoma: a multicentre, retrospective, longitudinal biomarker identification study with a nested case-control study. Lancet Oncol. 2015; 16:804-15. |
| 28 | Ren XW, Zhang JC. Changes of Plasma miR-29a Levels in Patients with Hepatocellular Carcinoma and Its Clinical Significance. J Mod Lab Med. 2017;32:33-5, 40. |
| 29 | Zheng JJ, Yu FJ, Dong PH, Bai YH, Chen BC. Expression of miRNA-29b and its clinical significances in primary hepatic carcinoma. Natl Med J China. 2013;93:888-91. |
| 30 | Liu C, Tang H, Deng L, Zhang J, Deng JP. Research of Diagnostic Application of Serum miRNA-1 8 6 and miRNA-30c in Patients with Hepatocellular Carcinoma. J Mod Lab Med. 2016;31:44-7. |
| 31 | Bhattacharya S, Steele R, Shrivastava S, Chakraborty S, Di Bisceglie AM, Ray RB. Serum miR-30e and miR-223 as Novel Noninvasive Biomarkers for Hepatocellular Carcinoma. Am J Pathol. 2016; 186:242-7. |
| 32 | Qiu LW, Wu W, Sai WL, Yang JL, Zhang HJ, Zheng WJ, Yao DF. Diagnostic application and comparative analysis of serum miR-96 and miR-182 in patients with hepatocellular carcinoma. Journal of Nantong University (Medical Sciences). 2014;358-61. |
| 33 | Xie Y, Yao Q, Butt AM, Guo J, Tian Z, Bao X, Li H, Meng Q, Lu J. Expression profiling of serum microRNA-101 in HBV-associated chronic hepatitis, liver cirrhosis, and hepatocellular carcinoma. Cancer Biol Ther. 2014; 15:1248-55. |
| 34 | Shaker O, Alhelf M, Morcos G, Elsharkawy A. miRNA-101-1 and miRNA-221 expressions and their polymorphisms as biomarkers for early diagnosis of hepatocellular carcinoma. Infect Genet Evol. 2017; 51:173-81. |
| 35 | Shi BM, Lu W, Ji K, Wang YF, Xiao S, Wang XY. Study on the value of serum miR-106b for the early diagnosis of hepatocellular carcinoma. World J Gastroenterol. 2017; 23:3713-20. |
| 36 | Jiang L, Li X, Cheng Q, Zhang BH. Plasma microRNA might as a potential biomarker for hepatocellular carcinoma and chronic liver disease screening. Tumour Biol. 2015; 36:7167-74. |
| 37 | Meng FL, Jia WD, Xu GL, Li JS, Wang W, Sun QK. Serum miR-106b expression and its clinical significance in hepatocellular carcinoma. Acta Univ Med Anhui. 2014;334-8. |
| 38 | Gui RF, Hu WH. The expression and significance of miRNAs in hepatocellular carcinoma serum. Anhui Med Pharm J. 2016;126-8. |
| 39 | Hung CH, Hu TH, Lu SN, Kuo FY, Chen CH, Wang JH, Huang CM, Lee CM, Lin CY, Yen YH, Chiu YC. Circulating microRNAs as biomarkers for diagnosis of early hepatocellular carcinoma associated with hepatitis B virus. Int J Cancer. 2016; 138:714-20. |
| 40 | Qi P, Cheng SQ, Wang H, Li N, Chen YF, Gao CF. Serum microRNAs as biomarkers for hepatocellular carcinoma in Chinese patients with chronic hepatitis B virus infection. PLoS One. 2011; 6:e28486. |
| 41 | Xu LN, Huang DF, Li F, Wei Q, Zhou GX, Zhang H. Expressions and significance of serum miRNA-122 and miRNA-221 in diagnosis of primary hepatic carcinoma. Jinagsu Medical Journal. 2015;41:1285-8. |
| 42 | Zhang Y, Tuo M, Liu SW. Vsalue of serum exosomes miRNA-122 detection in diagnosis of primary hepatocellular carcinoma. Lab Med Clin. 2017;14:1260-5. |
| 43 | Luo J, Chen M, Huang H, Yuan T, Zhang M, Zhang K, Deng S. Circulating microRNA-122a as a diagnostic marker for hepatocellular carcinoma. Onco Targets Ther. 2013; 6:577-83. |
| 44 | Zhang CH, Hu T, Yang XF, Li S, Lv X, He S, Yan HX, Tan YX, Chen RR, Wen ML, Li JC, Zuo JH. Role of circulating miRNA-122a and miRNA-6086 in diagnosis and prognosis of hepatocellular carcinoma. J Hunan Normal Univ (Med Sci). 2017;14:12-6. |
| 45 | Elemeery MN, Badr AN, Mohamed MA, Ghareeb DA. Validation of a serum microRNA panel as biomarkers for early diagnosis of hepatocellular carcinoma post-hepatitis C infection in Egyptian patients. World J Gastroenterol. 2017; 23:3864-75. |
| 46 | Chen S, Chen H, Gao S, Qiu S, Zhou H, Yu M, Tu J. Differential expression of plasma microRNA-125b in hepatitis B virus-related liver diseases and diagnostic potential for hepatitis B virus-induced hepatocellular carcinoma. Hepatol Res. 2017; 47:312-20. |
| 47 | Zuo D, Luo Y, Guo H, Zhang Ning. The diagnosis value of combined detection of serum mir-125b and alpha-fetoprotein for primary hepatocellular carcinoma. Chin J Clin Oncol. 2014;662-6. |
| 48 | Khairy A, Hamza I, Shaker O, Yosry A. Serum miRNA Panel in Egyptian Patients with Chronic Hepatitis C Related Hepatocellular Carcinoma. Asian Pac J Cancer Prev. 2016; 17:2699-703. |
| 49 | Ghosh A, Ghosh A, Datta S, Dasgupta D, Das S, Ray S, Gupta S, Datta S, Chowdhury A, Chatterjee R, Mohapatra SK, Banerjee S. Hepatic miR-126 is a potential plasma biomarker for detection of hepatitis B virus infected hepatocellular carcinoma. Int J Cancer. 2016; 138:2732-44. |
| 50 | Dhayat SA, Husing A, Senninger N, Schmidt HH, Haier J, Wolters H, Kabar I. Circulating microRNA-200 Family as Diagnostic Marker in Hepatocellular Carcinoma. PLoS One. 2015; 10:e140066. |
| 51 | Zhang J, Lin H, Wang XY, Zhang DQ, Chen JX, Zhuang Y, Zheng XL. Predictive value of microRNA-143 in evaluating the prognosis of patients with hepatocellular carcinoma. Cancer Biomark. 2017; 19:257-62. |
| 52 | Zhang ZQ, Meng H, Wang N, Liang LN, Liu LN, Lu SM, Luan Y. Serum microRNA 143 and microRNA 215 as potential biomarkers for the diagnosis of chronic hepatitis and hepatocellular carcinoma. Diagn Pathol. 2014; 9:135. |
| 53 | Xie Y, Tian Z, Qu YC, Bao XL, Zhang L, Lv J. Expression and clinical value of serum microRNA-143 in HBV associated liver diseases. Chin J Clinicians (Electronic Edition). 2013;11370-5. |
| 54 | Wang F, Ying H, He B, Pan Y, Sun H, Wang S. Circulating miR-148/152 family as potential biomarkers in hepatocellular carcinoma. Tumour Biol. 2016; 37:4945-53. |
| 55 | Yu F, Lu Z, Chen B, Dong P, Zheng J. microRNA-150: a promising novel biomarker for hepatitis B virus-related hepatocellular carcinoma. Diagn Pathol. 2015; 10:129. |
| 56 | Yang XM, Ren H, Wei R, Zhang X, Yang YM, Du LT, Zou MJ, Wang CX, Zhang Y. Expression level of serum circulating microRNA-152 and its clinical significance in HBV positive hepatocellular carcinoma. Chinese Journal of Current Advances in General Surgery. 2016;19:343-6. |
| 57 | Chen L, Chu F, Cao Y, Shao J, Wang F. Serum miR-182 and miR-331-3p as diagnostic and prognostic markers in patients with hepatocellular carcinoma. Tumour Biol. 2015; 36:7439-47. |
| 58 | Xia HN, Zhang M, Ye JJ, Chen P, Yue ZY. Serum miR-182 expression and its diagnostic value in hepatocellular cancer cells. Zhejiang Practical Medicine. 2015;191-8. |
| 59 | Wang Y, Liang Z, Gao Y, Zhai D, Rao Q, Shi W, Yang B, Jing L, Guo H, Liu T, Liu J, DU Z. Factors influencing circulating MicroRNA level in the studies of hepatocellular carcinoma biomarker. Neoplasma. 2015; 62:798-804. |
| 60 | Yin J, Hou P, Wu Z, Wang T, Nie Y. Circulating miR-375 and miR-199a-3p as potential biomarkers for the diagnosis of hepatocellular carcinoma. Tumour Biol. 2015; 36:4501-7. |
| 61 | Mi XG, Liu L, Li SQ, Fang YQ. Value of serum microRNA-199a/b-3p on early diagnosis of hepatocellular carci-noma. Chin J Immun. 2015;683-9. |
| 62 | Yu L, Zhang KJ, Chai L, Li BL, Yang ZH, Liu JB. Expression changes of miR-202 in cancerous tissues and serum of patients with primary hepatocellular carcinoma. Shandong Medical Journal. 2017;57:1-4. |
| 63 | Lu CY, Chen SY, Peng HL, Kan PY, Chang WC, Yen CJ. Cell-free methylation markers with diagnostic and prognostic potential in hepatocellular carcinoma. Oncotarget. 2017; 8:6406-18. |
| 64 | Chen SS, Chen H, Gao SS, Zhou H, Qiu SL, Yu MX, Tu JC. Differential Expression of Plasma miR-205 in HBV-Related Liver Diseases and Diagnostic Potential for HBV-Induced Hepatocellular Carcinoma. Medical Journal of Wuhan University. 2016;37:445-50. |
| 65 | Wu MF, Zhu JH, Wu XM. Expression and clinical value of serum miR-205-5p in HBV-related hepatocellular carcinoma. Journal of Tropical Medicine. 2016;31-4. |
| 66 | Yang L, Xu Q, Xie H, Gu G, Jiang J. Expression of serum miR-218 in hepatocellular carcinoma and its prognostic significance. Clin Transl Oncol. 2016; 18:841-7. |
| 67 | El-Garem H, Ammer A, Shehab H, Shaker O, Anwer M, El-Akel W, Omar H. Circulating microRNA, miR-122 and miR-221 signature in Egyptian patients with chronic hepatitis C related hepatocellular carcinoma. World J Hepatol. 2014; 6:818-24. |
| 68 | Yang ZF, Chen H, Jiang XM. Serum miR-221 and miR-338 expression and its clinical significance in hepatocellular carcinoma. Guangdong Medical Journal. 2014;841-3. |
| 69 | Motawi TM, Sadik NA, Shaker OG, Ghaleb MH. Elevated serum microRNA-122/222 levels are potential diagnostic biomarkers in Egyptian patients with chronic hepatitis C but not hepatic cancer. Tumour Biol. 2016; 37:9865-74. |
| 70 | Okajima W, Komatsu S, Ichikawa D, Miyamae M, Kawaguchi T, Hirajima S, Ohashi T, Imamura T, Kiuchi J, Arita T, Konishi H, Shiozaki A, Moriumura R, et al. Circulating microRNA profiles in plasma: identification of miR-224 as a novel diagnostic biomarker in hepatocellular carcinoma independent of hepatic function. Oncotarget. 2016; 7:53820-36. |
| 71 | Lin L, Lu B, Yu J, Liu W, Zhou A. Serum miR-224 as a biomarker for detection of hepatocellular carcinoma at early stage. Clin Res Hepatol Gastroenterol. 2016; 40:397-404. |
| 72 | Zhang H, Chen XY, Yang B, Zhang MH, Wang RJ. Research on the serum level of microRNA-224 in hepatocellular carcinoma patients and its clinical diagnostic significance. Chin J Clin Oncol. 2014;576-9. |
| 73 | Lin L, Lu BC. Value of miR-224 for detection of HCC at early stage. Chinese Journal of General Practice. 2016;14:1445-8. |
| 74 | He K, Hu Z, Ruan J, Ma Q, Zhong F, Cheng X, Sun S, Zhou J. MicroRNA301 is a potential diagnostic biomarker for hepatocellular cancer. Int J Clin Exp Pathol. 2015; 8:5603-8. |
| 75 | Zhang Y, Shao JG, Chen L, Bian ZL, Guan HT. Evaluate the significance of serum miR-335 in the diagnosis of hepatocellular carcinoma. Journal of Nantong University (Medical Sciences). 2016;36:174-8. |
| 76 | Pei LL, Ren WH, Li JS, Xu GL, Jia WD, Ma JL. The expression and significance of four miRNAs in hepatocellular carcinoma serum. Acta Univ Med Anhui. 2014;1287-91. |
| 77 | Shen J, Wang A, Wang Q, Gurvich I, Siegel AB, Remotti H, Santella RM. Exploration of genome-wide circulating microRNA in hepatocellular carcinoma: MiR-483-5p as a potential biomarker. Cancer Epidemiol Biomarkers Prev. 2013; 22:2364-73. |
| 78 | Zhang Z, Ge S, Wang X, Yuan Q, Yan Q, Ye H, Che Y, Lin Y, Zhang J, Liu P. Serum miR-483-5p as a potential biomarker to detect hepatocellular carcinoma. Hepatol Int. 2013; 7:199-207. |
| 79 | Fornari F, Ferracin M, Trere D, Milazzo M, Marinelli S, Galassi M, Venerandi L, Pollutri D, Patrizi C, Borghi A, Foschi FG, Stefanini GF, Negrini M, et al. Circulating microRNAs, miR-939, miR-595, miR-519d and miR-494, Identify Cirrhotic Patients with HCC. PLoS One. 2015; 10:e141448. |
| 80 | Xue YJ, Chen J, Shi W, Zhang JY, Zhang H, Ju SQ. Expression and its significance of serum microRNA-574-3p in hepatitis B virus-related hepatocellular carcinoma. Chin J Dig. 2014;34:732-6. |
| 81 | Hu T, Li JC, Yang XF, Zhang CH, Li S, Lv X, He S, Yan HX. Study of circulating microRNA-4281 for diagnosis and prognostic valuation in hepatocellular carcinoma. J Univ South China (Med Ed). 2017;45:57-60. |
| 82 | Wu XM, Xi ZF, Liao P, Huang HD, Huang XY, Wang C, Ma Y, Xia Q, Yao JG, Long XD. Diagnostic and prognostic potential of serum microRNA-4651 for patients with hepatocellular carcinoma related to aflatoxin B1. Oncotarget. 2017; 8:81235-49. |
