# Supplementary material for: Serum microRNA signatures and metabolomics have high diagnostic value in hepatocellular carcinoma
Source: Oncotarget. 2017 Nov 1;8(65):108810–24. doi: 10.18632/oncotarget.22224 (PMC5752483; doi:10.18632/oncotarget.22224)
Supplement: Supplementary file 3 [file oncotarget-08-108810-s003.docx]

**Supplementary Table 2: Characteristics of the included studies**

| **Article number** | **First author** | **Published year** | **Country** | **Ethnicity** | **HCC group** | | | **Control group** | | | **Specimen** | **MicroRNA** | **Reference RNA** |
| --- | --- | --- | --- | --- | --- | --- | --- | --- | --- | --- | --- | --- | --- |
|  |  |  |  |  | **Sample size** | **Mean age** | **Gender** | **Sample size** | **Mean age** | **Gender** |  |  |  |
| 1 | Tarek M | 2017 | Egypt | Caucasian | 30 | / | 23/7 | 60 | / | 32/18 | Serum | miR-7 | SNORD68 |
| 2 | Jiang L | 2015 | China | Asian | 27 | 51.7 | 15/12 | 81 | 50.1 | 44/37 | Serum | miR-10b, miR-106b, miR-181a | U6 |
| 3 | Liu AM | 2012 | China | Asian | 57 | / | 49/8 | 59 | / | 42/17 | Serum | miR-15b, miR-21, miR-130b, miR-183 | (Absolute quantification) |
| 4 | Chen Y | 2015 | China | Asian | 37 | / | 44/3 | 60 | / | 30/30 | Plasma | miR-15b-5p, miR-338-5p, miR-764 | miR-16 |
| 5 | Chen Y | 2015 | China | Asian | 39 | / | 36/3 | 57 | / | 32/25 | Plasma | miR-15b-5p, miR-21-5p, miR-338-5p | miR-16 |
| 6 | El-Abd NE | 2015 | Egypt | Caucasian | 40 | 56.5 | 27/13 | 40 | 56.4 | 23/17 | Serum | miR-16 | RNU48 |
| 7 | El-Tawdi AH | 2016 | Egypt | Caucasian | 78 | / | 56/22 | 78 | / | 66/12 | Serum | miR-16-2, miR-21 | U6 |
| 8 | Zhang Y | 2017 | China | Asian | 115 | 55.2 | 86/29 | 40 | 54.4 | 25/15 | Serum | miR-16-2-3p, miR-92a-3p, miR-107, miR-3126-5p | U6 |
| 9 | Li L | 2012 | China | Asian | 101 | 54 | 76/25 | 90 | 51.7 | 69/21 | Serum | miR-18a | U6 |
| 10 | Motawi TK | 2015 | Egypt | Caucasian | 112 | 60 | 92/20 | 167 | 44.3 | 123/44 | Serum | miR-19a, miR-34a, miR-130a, miR-146a, miR-192, miR-195, miR-296 | SNORD68 |
| 11 | Wen Y | 2015 | China | Asian | 67 | 48.5 | 46/21 | 82 | 48.6 | 59/23 | Plasma | miR-20a-5p, miR-25-3p, miR-30a-5p, miR-92a-3p, miR-132-3p, miR-185-5p, miR-320a, miR-324-3p | cel-miR-39 |
| 12 | Guo X | 2017 | China | Asian | 175 | 54.3 | 97/78 | 278 | 50 | 161/117 | Serum | miR-21 | U6 |
| 13 | Zhuang C | 2016 | China | Asian | 52 | 51.1 | 40/12 | 85 | 50.4 | 54/31 | Serum | miR-21, miR-26a, miR-101 | U6 |
| 14 | Amr KS | 2016 | Egypt | Caucasian | 23 | 54.3 | 22/1 | 17 | 45.2 | 15/2 | Serum | miR-21, miR-199a | RNU48 |
| 15 | Tomimaru Y | 2012 | Japan | Asian | 126 | 63 | 99/27 | 80 | 62 | 57/23 | Plasma | miR-21 | miR-16 |
| 16 | Xu J | 2011 | China | Asian | 101 | / | 78/23 | 89 | / | 68/21 | Serum | miR-21, miR-122, miR-223 | RNU48, U6 |
| 17 | Chen DD | 2016 | China | Asian | 46 | 49.2 | 26/20 | 93 | 45.7 | 49/44 | Serum | miR-21, miR-192 | / |
| 18 | Gao L | 2015 | China | Asian | 60 | 54.2 | 41/19 | 123 | / | / | Plasma | miR-21 | miR-16 |
| 19 | Qin ZH | 2013 | China | Asian | 55 | 64 | 30/25 | 110 | 54.4 | 55/55 | Plasma | miR-21 | miR-16 |
| 20 | Wang P | 2017 | China | Asian | 69 | 58.8 | 51/18 | 87 | 54.5 | 60/27 | Serum | miR-21, miR-4429 | (Absolute quantification) |
| 21 | Chen XH | 2016 | China | Asian | 62 | 51.3 | 49/13 | 62 | 50.5 | 46/16 | Serum | miR-21 | miR-16 |
| 22 | Meng FL | 2014 | China | Asian | 72 | / | 57/15 | 31 | / | / | Serum | miR-24-3p | cel-miR-39 |
| 23 | Zuo D | 2016 | China | Asian | 90 | 54.7 | 68/22 | 60 | 49.2 | 24/36 | Serum | miR-26a, miR-27a, miR-125b, miR-223 | U6 |
| 24 | Tan Y | 2014 | China | Asian | 135 | 53.6 | 112/23 | 222 | 40.6 | 152/70 | Serum | miR-26a-5p, miR-122-5p, miR-141-3p, miR-192-5p, miR-199a-5p, miR-206, miR-433-5p, miR-1228-5p | miR-24 |
| 25 | Chen HY | 2016 | China | Asian | 50 | / | 35/15 | 50 | / | 32/18 | Blood | miR-26b | U6 |
| 26 | Zhu HT | 2017 | China | Asian | 91 | 51 | 78/13 | 91 | 40.9 | 56/35 | Serum | miR-27b-3p, miR-192-5p | cel-miR-39 |
| 27 | Lin XJ | 2015 | China | Asian | 108 | 47.5 | 90/18 | 149 | 45.4 | 108/41 | Serum | miR-29a, miR-29c, miR-133a, miR-143, miR-145, miR-192, miR-505 | cel-miR-67 |
| 28 | Ren XW | 2017 | China | Asian | 30 | 57 | 25/5 | 60 | 39.5 | 39/21 | Plasma | miR-29a | cel-miR-39 |
| 29 | Zheng JJ | 2013 | China | Asian | 87 | 51.7 | 58/29 | 96 | 56.2 | 52/44 | Serum | miR-29b | U6 |
| 30 | Liu C | 2016 | China | Asian | 55 | 56.4 | 38/17 | 110 | / | / | Serum | miR-30c, miR-186 | U6 |
| 31 | Bhattacharya S | 2016 | USA | Caucasian | 39 | 59.3 | 29/10 | 31 | 50.1 | 23/8 | Serum | miR-30e, miR-223 | U6 |
| 32 | Qiu LW | 2014 | China | Asian | 60 | 56.4 | 42/18 | 180 | / | 120/60 | Serum | miR-96, miR-182 | miR-16 |
| 33 | Xie Y | 2014 | China | Asian | 67 | 51.7 | 57/10 | 170 | 40 | 118/52 | Serum | miR-101 | cel-miR-39 |
| 34 | Shaker O | 2017 | Egypt | Caucasian | 37 | / | 29/8 | 78 | / | 53/25 | Serum | miR-101-1, miR-221 | SNORD68 |
| 35 | Shi BM | 2017 | China | Asian | 35 | / | / | 300 | / | / | Serum | miR-106b | cel-miR-39 |
| 36 | Jiang L | 2015 | China | Asian | 47 | 56.1 | 36/11 | 61 | 53.8 | 46/15 | Plasma | miR-106b | U6 |
| 37 | Meng FL | 2014 | China | Asian | 47 | 55.4 | 37/10 | 53 | 52.5 | 41/12 | Serum | miR-106b | cel-miR-39 |
| 38 | Gui RF | 2016 | China | Asian | 50 | 51.4 | 32/18 | 100 | 53.5 | 69/31 | Serum | miR-106b | miR-16 |
| 39 | Hung CH | 2016 | China | Asian | 120 | 58.5 | 96/24 | 30 | 60.3 | 24/6 | Serum | miR-122, miR-let-7b | U6 |
| 40 | Qi P | 2011 | China | Asian | 70 | 49 | 55/15 | 82 | 42.1 | 61/21 | Serum | miR-122 | miR-16 |
| 41 | Xu LN | 2015 | China | Asian | 45 | / | 37/8 | 50 | / | 30/20 | Serum | miR-122, miR-221 | U6 |
| 42 | Zhang Y | 2017 | China | Asian | 82 | 49.2 | 48/34 | 79 | 45.4 | 43/36 | Serum | miR-122 | U6 |
| 43 | Luo J | 2013 | China | Asian | 85 | 53.6 | 70/15 | 85 | 50.8 | 69/16 | Serum | miR-122a | U6 |
| 44 | Zhang CH | 2017 | China | Asian | 45 | / | / | 45 | / | / | Serum | miR-122a, miR-6086 | / |
| 45 | Elemeery MN | 2017 | Egypt | Caucasian | 224 | 55 | / | 334 | 42.5 | / | Serum | miR-125b, miR-138b, miR-145, miR-214-5p, miR-375, miR-494, miR-1269 | SNORD68 |
| 46 | Chen S | 2017 | China | Asian | 64 | 54 | 53/11 | 178 | 53.2 | 134/44 | Plasma | miR-125b | U6 |
| 47 | Zuo D | 2014 | China | Asian | 65 | / | 48/17 | 30 | / | 17/13 | Serum | miR-125b | U6 |
| 48 | Khairy A | 2016 | Egypt | Caucasian | 23 | 59.5 | 20/3 | 55 | 46.1 | 39/16 | Serum | miR-126, miR-129, miR-155, miR-203, miR-223 | SNORD68 |
| 49 | Ghosh A | 2016 | India | Caucasian | 59 | 46.2 | 50/9 | 48 | 38.9 | 38/10 | Plasma | miR-126, miR-142-3p | cel-miR-39 |
| 50 | Dhayat SA | 2015 | Germany | Caucasian | 22 | 64 | 20/2 | 37 | 42.8 | 20/17 | Serum | miR-141, miR-200a | cel-miR-39 |
| 51 | Zhang J | 2017 | China | Asian | 131 | 50.5 | 88/43 | 122 | 49.4 | 79/43 | Serum | miR-143 | GAPDH |
| 52 | Zhang ZQ | 2014 | China | Asian | 95 | 54.2 | 60/35 | 127 | 52.6 | 71/56 | Serum | miR-143, miR-215 | U6 |
| 53 | Xie Y | 2013 | China | Asian | 67 | 51.5 | 57/10 | 30 | 35.5 | 21/9 | Serum | miR-143 | cel-miR-67, cel-miR-356 |
| 54 | Wang F | 2016 | China | Asian | 76 | / | 66/10 | 117 | / | / | Serum | miR-148a, miR-148b, miR-152 | cel-miR-39 |
| 55 | Yu F | 2015 | China | Asian | 120 | 58 | 75/45 | 230 | 52.4 | 132/98 | Serum | miR-150 | miR-16 |
| 56 | Yang XM | 2016 | China | Asian | 36 | 53.7 | 30/6 | 28 | 62.6 | 20/8 | Serum | miR-152 | U6 |
| 57 | Chen L | 2015 | China | Asian | 103 | 52 | 89/14 | 95 | 50 | 80/15 | Serum | miR-182, miR-331-3p | U6 |
| 58 | Xia HN | 2015 | China | Asian | 40 | 48 | 29/11 | 40 | 45 | 27/13 | Plasma | miR-182 | U6 |
| 59 | Wang Y | 2015 | China | Asian | 38 | / | / | 52 | / | / | Plasma | miR-183 | cel-miR-39 |
| 60 | Yin J | 2015 | China | Asian | 78 | 56.3 | 49/28 | 156 | 55.8 | 102/54 | Serum | miR-199-3p, miR-375 | U6 |
| 61 | Mi XG | 2015 | China | Asian | 30 | 52.8 | 21/9 | 60 | 49 | 40/20 | Serum | miR-199a/b-3p | U6 |
| 62 | Yu L | 2017 | China | Asian | 70 | 48 | 59/11 | 30 | 38 | 18/12 | Serum | miR-202 | U6 |
| 63 | Lu CY | 2017 | China | Asian | 84 | / | / | 103 | / | / | Plasma | miR-203 | β-actin |
| 64 | Chen SS | 2016 | China | Asian | 64 | 54 | 53/11 | 115 | 53 | 83/32 | Plasma | miR-205 | U6 |
| 65 | Wu MF | 2016 | China | Asian | 34 | 45.1 | 28/6 | 60 | 49.6 | / | Serum | miR-205-5p | U6 |
| 66 | Yang L | 2016 | China | Asian | 156 | 53.7 | 111/45 | 162 | / | / | Serum | miR-218 | U6 |
| 67 | El-Garem H | 2014 | Egypt | Caucasian | 30 | 60.3 | 25/5 | 60 | 46.6 | 43/17 | Serum | miR-221 | SNORD68 |
| 68 | Yang ZF | 2014 | China | Asian | 80 | 52 | 48/32 | 140 | 39.3 | 79/61 | Serum | miR-221, miR-338 | U6 |
| 69 | Motawi TM | 2016 | Egypt | Caucasian | 60 | 60.3 | 50/10 | 40 | 43 | 28/12 | Serum | miR-222 | SNORD68 |
| 70 | Okajima W | 2016 | Japan | Asian | 87 | / | 59/28 | 55 | / | / | Plasma | miR-224 | cel-miR-39 |
| 71 | Lin L | 2016 | China | Asian | 122 | 51.4 | 92/30 | 213 | 47.6 | 152/61 | Serum | miR-224 | cel-miR-39 |
| 72 | Zhang H | 2014 | China | Asian | 42 | 53.2 | 24/18 | 131 | / | / | Serum | miR-224 | U6 |
| 73 | Lin L | 2016 | China | Asian | 96 | 56 | 72/24 | 146 | 47.6 | 105/41 | Serum | miR-224 | cel-miR-39 |
| 74 | He K | 2015 | China | Asian | 42 | / | 30/12 | 38 | / | / | Serum | miR-301 | GAPDH |
| 75 | Zhang Y | 2016 | China | Asian | 50 | 56 | 36/14 | 40 | 50 | 20/20 | Serum | miR-335 | U6 |
| 76 | Pei LL | 2014 | China | Asian | 66 | 54.1 | 50/16 | 40 | 52.2 | 32/8 | Serum | miR-451a | miR-16 |
| 77 | Shen J | 2013 | USA | Caucasian | 49 | 61.1 | 41/8 | 49 | 61.5 | 41/8 | Plasma | miR-483-5p | cel-miR-39 |
| 78 | Zhang Z | 2013 | China | Asian | 112 | 54.4 | 80/32 | 141 | 54.5 | 100/41 | Serum | miR-483-5p, miR-500a | (Absolute quantification) |
| 79 | Fornari F | 2015 | Italy | Caucasian | 87 | / | / | 31 | / | / | Serum | miR-519d, miR-595, miR-939 | cel-miR-39 |
| 80 | Xue YJ | 2014 | China | Asian | 90 | 50 | 55/35 | 90 | 47.7 | 52/38 | Serum | miR-574-3p | U6 |
| 81 | Hu T | 2017 | China | Asian | 45 | 57.6 | 33/12 | 45 | 45.2 | / | Serum | miR-4281 | cel-miR-39 |
| 82 | Wu XM | 2017 | China | Asian | 279 | 49.9 | 193/86 | 662 | 460/202 | 49.8 | Serum | miR-4651 | cel-miR-39 |

Abbreviations: HCC, hepatocellular carcinoma.
